# Supplementary figures and images for: Implications of Hereditary Origin on the Immune Phenotype of Mismatch Repair-Deficient Cancers: Systematic Literature Review
Source: J Clin Med. 2020 Jun 4;9(6):1741. doi: 10.3390/jcm9061741 (PMC7357024; doi:10.3390/jcm9061741)

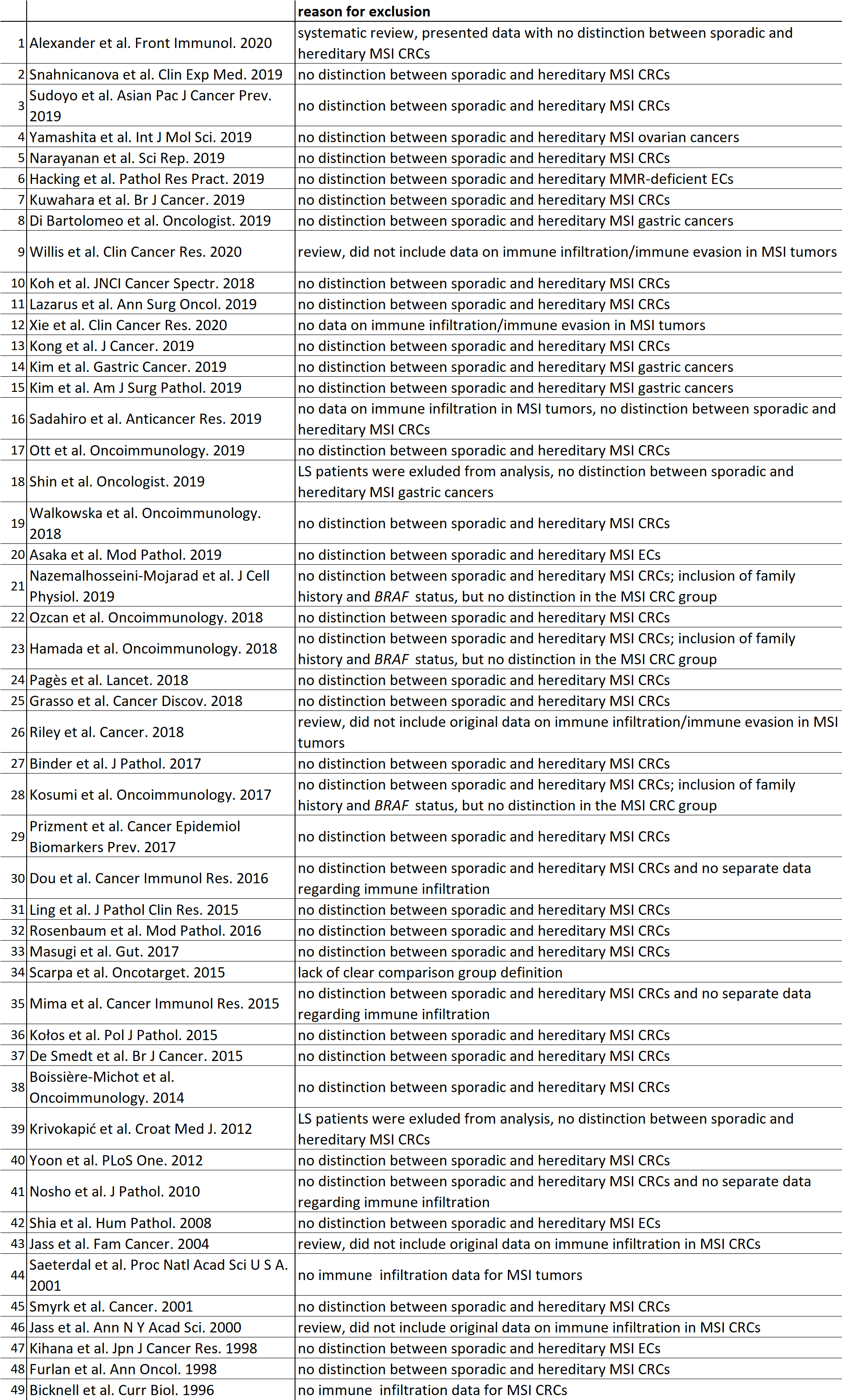

Supplement: Supplementary file 1 [file jcm-09-01741-s001.zip › supplementary_jcm-813082_030620/TableS1.png]
